# “A patchwork of services” – caring for women who sustain severe perineal trauma in New South Wales – from the perspective of women and midwives

**DOI:** 10.1186/1471-2393-14-236

**Published:** 2014-07-18

**Authors:** Holly S Priddis, Virginia Schmied, Christine Kettle, Anne Sneddon, Hannah G Dahlen

**Affiliations:** 1 School of Nursing and Midwifery, University of Western Sydney, Building EB, Parramatta Campus, Locked Bag 1797, Penrith South DC NSW 2751, Australia; 2 Staffordshire University, Blackheath Lane, Stafford, Staffordshire ST18 0AD, UK; 3 Obstetrics and Gynaecology Gold Coast, Griffith University, Gold Coast, Australia

**Keywords:** Qualitative research, Severe perineal trauma, Health services, Birth

## Abstract

**Background:**

Current research into severe perineal trauma (3^rd^ and 4^th^ degree) focuses upon identification of risk factors, preventative practices and methods of repair, with little focus on women’s experiences of, and interactions with, health professionals following severe perineal trauma (SPT). The aim of this study is to describe current health services provided to women in New South Wales (NSW) who have experienced SPT from the perspective of Clinical Midwifery Consultants (CMC) and women.

**Methods:**

This study used a descriptive qualitative design and reports on the findings of a component of a larger mixed methods study. Data were collected through a semi-structured discussion group using a variety of non-directive, open-ended questions leading CMCs of NSW. A survey was distributed prior to the discussion group to collect further information and enable a more comprehensive understanding of services provided. Data from individual interviews with twelve women who had experienced SPT during vaginal birth is used to provide greater insight into their interactions with, and ease of access to, health service providers in NSW. An integrative approach was undertaken in reporting the findings which involved comparing and analysing findings from the three sets of data.

**Results:**

One overarching theme was identified: *A Patchwork of Policy and Process* which identified that current health services operate in a ‘patchwork’ manner when caring for women who sustain SPT. They are characterised by lack of consistency in practice and standardisation of care. Within the overarching theme, four subthemes were identified: *Falling through the gaps; Qualifications, skills and attitudes of health professionals; Caring for women who have sustained SPT; and Gold standard care: how would it look?*

**Conclusion:**

The findings from this study suggest that current health services in NSW represent a ‘patchwork’ of service provision for women who have sustained SPT. It appeared that women seek compassionate and supportive care based upon a clear exchange of information, and this should be considered when reflecting upon health service design. This study highlights the benefits of establishing multi-disciplinary collaborative specialist clinics to support women who experience SPT and associated morbidities, with the aim of providing comprehensive physiological and psychological support.

## Background

During vaginal birth, approximately two thirds of Australian women will experience some degree of trauma to the perineum [1]. Perineal trauma is defined by the extent of injury sustained to the perineum, skin, perineal muscles, labia, clitoris, urethra and anal sphincter. It is estimated that approximately 1.8% will experience severe perineal trauma (SPT), which is defined as a third or fourth degree tear to the perineum and anal sphincter complex (Table 1), and may result in both short and long term physical and emotional morbidities for women [2,3]. A comparison of data reporting upon the incidence of SPT in New South Wales (NSW) from 2000–2008 found a significant increase in the overall rate of SPT from 1.4% to 1.9% [4]. This increase was particularly seen in third degree tears and extensions following episiotomies [4]. Internationally, the incidence of SPT is reported to range from between 0.5 – 7%. This variation may be due to the reporting process, obstetric management, and differences in the definition of perineal trauma [5-7].

**Table 1:** **Definition of perineal trauma **[2]

**Degree**	**Trauma to Perineum**
First degree	Laceration of vaginal epithelium or perineal skin
Second degree	Involvement of perineal muscles, not anal sphincter
Third degree	Disruption of anal sphincter muscles –
	3a: <50% external sphincter torn
	3b: >50% external sphincter torn
	3c: external and internal sphincter torn
Fourth degree	Third degree tear with rupture of trauma to anal epithelium

Current research into SPT focusses upon the identification of risk factors, preventative practices and the most appropriate methods of repair, with little focus on women’s experiences of, and interactions with, health professionals following SPT [8-10]. In Australia, care provided to women in the first week following childbirth is most often provided by a midwife either in hospital or in the community during home visits. For women who are cared for within the private model, postnatal support is provided by an obstetrician. The average duration of care is two to ten days postnatal, however some models of maternity care provide support up to six weeks following the birth [11].

Women have reported variation in information received regarding the degree of perineal trauma they sustained, and the symptoms that may develop varied, contributing to feelings of vulnerability and abandonment [8]. Access to specialised postnatal care may be beneficial for women’s long term physical and psychological wellbeing, however current health services do not appear to provide adequate support nor address the physical and psychological needs of women [12]. The actions of health care providers, and how they interact with women during the time of birth, has an impact upon how women understand and experience SPT and at times these interactions have been reported as being insensitive and inappropriate [9,13]. Previous research indicates that women can feel vulnerable, exposed and abandoned throughout the labour and birth and suturing process, and that these feelings may be influenced by the actions of health care providers [8]. Research reporting on the experiences of health care providers caring for women who sustain SPT, focusses upon their ability to identify and repair perineal trauma, and the psychological impact of caring for a woman who sustain a childbirth related injury [14,15]. However, there appears to be a lack of literature describing the structure of health service provision for women who sustains SPT and related co-morbidities from the perspective of clinical leaders.

The aim of this study is to describe current health service in NSW provided to women with SPT from the perspective of Clinical Midwifery Consultants and women.

## Method

This study used a descriptive qualitative design [16-18]. Data were collected through a discussion group with the Clinical Midwifery Consultants (CMC’s) of NSW. Data from individual interviews with women who had sustained SPT is used to provide greater insight into their interactions with, and ease of access to, health service providers in NSW.

This paper reports on the findings of a component of a larger mixed methods study which included four phases: A meta-ethnographic study examining the experiences for women who had sustained a postpartum physical morbidity including SPT [8]; a linked data study which reported upon the risk of recurrence, subsequent mode of birth and morbidity for women who experienced SPT in NSW between June 2000 and July 2008 [19]; In-depth interviews to explore the experiences of women who had sustained SPT [12]; and a discussion group and survey conducted with the Clinical Midwifery Consultants of NSW which is reported on in this paper and integrated with relevant data quotes from interviews with women related to interactions with, and service provision from, health care providers which were not published in the previous study.

### Participants and recruitment

Fourteen CMCs participated in this study. All CMCs were experienced midwives currently practising in NSW and in their role as a consultant they are responsible for writing policy, supporting best practice, and providing midwifery leadership across the state. They are ideally positioned within their roles to have a comprehensive view of how the service works and where the strengths and weaknesses lie. This group of CMCs meet quarterly at a Sydney location.

Twelve women who had sustained SPT were interviewed for this study, the recruitment and participation process has been previously published [12]. Data not published in the previous study in relation to service provision for SPT is used in this study to contrast or support the CMC’s comments.

To ensure informed consent for both the participants of the discussion group and the women who were interviewed, participation was self-determined in response to an information sheet distributed via email which provided full disclosure of the research currently being undertaken. Each woman who contacted the researcher enquiring about the research who met the criteria for recruitment was provided with the information sheet. Participants were given the contact details of the researcher to ask any questions prior to giving consent. Participants were then asked to read and sign the consent form if they agreed to participate. Both the women and the CMCs were assured of confidentiality and advised that all data would be de-identified during transcription of the recording to protect identities. It was further clarified that participation was optional, that participants were able to request that digital recordings cease immediately at any time during the discussion group session, and were given the opportunity of withdrawing from participation at any time without penalty [20].

### Data collection

#### ***Discussion group with the CMCs***

The purpose of the discussion group was to develop an understanding of current health services provisions in NSW for women who sustain SPT as reported by currently practicing CMC’s working within the NSW Health system. The discussion group was one hour in duration, recorded with permission via signed consent using a digital voice recorder, and was transcribed verbatim by an external transcription company. The discussion group was facilitated by the first and last authors and was semi-structured, using a variety of non-directive open-ended questions which were used as triggers to stimulate conversation regarding the topic at hand (Hammersley & Atkinson, 2007).

#### ***Survey of CMCs***

Due to the size of the group and the limited time frame allocated (one hour) this did not lend itself to the level of interaction possible in a smaller discussion group [21,22]. Therefore a survey, containing nine multiple choice questions and one short answer response, was distributed to all participants to collect further information and enable a more comprehensive understanding of the services provided in NSW (Tables 2 and 3). The questions for the discussion group and survey were designed in response to the overarching themes and findings that were identified in previously conducted research which has been reported [12,19].

**Table 2:** CMCs responses to survey questions

	**Questions asked in survey of CMCs**	**Responses (number)**
	In what location are the majority of third degree perineal tears repaired?	
	a Birthing room	8
	b Theatre	5
	c Other	1
		(3 respondents included two locations in their response)
	In what location are the majority of fourth degree perineal tears repaired?	
	a Birthing room	2
	b Theatre	11
	c Other	1
	What suture material is predominantly used for severe perineal trauma repairs?	
	a Vicryl	8
	b Dexon	3
	c Catgut	3
	d Polysorb	0
	e Other	0
	What method of suturing is predominantly undertaken?	
	a Overlapping	1
	b End to end	1
	c Don’t know	2
	d Different with different practitioners	8
		(one respondent answered with Figure Eight)
	Who is permitted to repair severe perineal trauma?	
	a Midwives	1
	b Residents	9
	c Registrars	3
	d Other	0
		(6 respondents included two health professionals in their response)
	When severe perineal trauma occurs is it a reportable incident?	
	Yes	12
	No	1
	Sometimes	1
	Is there a follow up clinic available that women are referred to?	
	a Yes	10
	b No	3
		(one respondent answered with “unsure”)
	Has a recent audit been undertaken on the incidence of severe perineal trauma within your area/unit?	
	a Yes	10
	b No	3
		(one respondent answered with “unsure”)
	Do you think the incidence of third and/or fourth degree perineal trauma is rising?	
	a Yes	10
	b No	2
	c Unsure	2

**Table 3:** Survey question 10: Rank in order from 1 to 10 (1 being most significant, 10 being least significant) what you feel contributes to the incidence of severe perineal trauma

**Item**	**Average score**
Position for second stage	2.08
Instrumental birth	3.38
Episiotomy	3.46
Parity	5.81
Hands off technique	6.1
Ethnicity of woman	6.25
Position for labour	6.36
Model of care	6.36
Epidural	6.54
Maternal weight	7.36
Maternal age	7.72
Gender of baby	10

#### ***Interviews with women***

In addition, data were collected in face to face interviews with twelve women who had experienced SPT following vaginal birth. The purpose of the interviews was to explore the way women experienced and understood SPT, and their interactions and experiences with health professionals and health services. Each interview was between one to two hours duration and was semi-structured, using open ended questions [23]. The methods used for these interviews have been fully described in a previous paper [12]. Pseudonyms are used for the participants throughout this paper to protect their identity.

#### ***Data analysis***

An integrative approach has been used in reporting the findings which has involved comparing and analysing the findings from the three sets of data [24]. Integration was undertaken with the aim of facilitating a greater understanding of the topic under investigation [24,25]. Initially, the analysis of both datasets from the CMC discussion group and the interviews with women were conducted separately using thematic analysis. Through the process of thematic analysis, the first author individually read the transcript from both the interviews and the discussion group to identify patterns, of words or statements, that related to the focus of the study [26]. These groups were then placed in broad categories, and these results were discussed with the co-authors [27]. The broad categories were then analysed in detail to identify subthemes or key concepts which represented the patterns within the broad categories. Overarching themes were then developed to accurately reflect the findings within the data [12,26].

The survey responses were recorded and compared with the themes identified from the CMC discussion group, with similarities and disparities noted. The priority data set represented in this paper are the themes which were identified following the discussion group with the CMCs, the words of the women have been incorporated to provide added depth to the main themes. The focus of discussion with the CMCs was not always represented in conversations with the women and therefore there was no comparative data to include. During comparative analysis, the major themes and subthemes arising from the two separate datasets were analysed and compared to determine similarities and differences. The themes that overlapped or were common to both data sets were then integrated to undertake a second level of analysis. Through this process common themes were identified which enabled an understanding of how health services in NSW care for women who sustain SPT from the perspective of women and CMC’s.

Ethics approval was obtained by the University of Western Sydney Human Research Ethics Committee, approval number H9298.

## Results

### Participant demographics

The CMC participants represented both regional and rural local health districts (LHD), and individual hospitals and maternity services, across NSW. At the time of this research there were 16 LHD’s in NSW, all but one LHD was represented in this discussion group. All CMC’s who participated were female, with an average age of 52 years (Table 4).

**Table 4:** CMC demographics

**CMCs**	**Demographics**
Total participants	14
Average age in years	52.07 years
Average years of practice	30.8 years
Average years in current role	11.8 years
Australian born	9
Aboriginal or Torres Strait Islander	0
Other	5

The demographics for the women who participated in this study are presented in Table 5.

**Table 5:** Demographics of women participants

**Women**	**Demographics**
Total participants	12
Average age in years	35
Primiparous	5
Multiparous	7
Australian born	9
ATSI	0
Other	3
Educational background:	
Uni degree	7
Diploma or equivalent	3
HSC	2
Degree of trauma:	
3^rd^	11
4^th^	1
Model of care:	
Standard	5
Caseload	1
Private midwife	3
Group midwifery	1
Private obstetric	2

### Themes and subthemes

**Figure 1:**
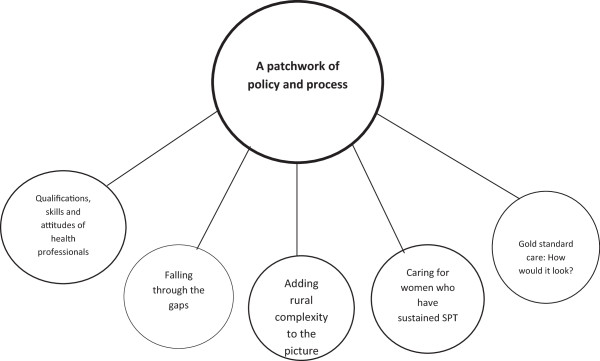
One diagrammatic representation of themes.

One overarching theme was identified following the process of comparative analysis: *A patchwork of policy and process*. Within the overarching theme, four subthemes were identified: *Falling through the gaps; Qualifications, skills and attitudes of health professionals; Caring for women who have sustained SPT; and Gold standard care: how would it look?* (Figure 1).

#### ***Overarching theme: a patchwork of policy and process***

The overarching theme that emerged from the discussion group was that health services in NSW operate within a patchwork of policy and processes when providing care for women who sustain SPT. The CMC participants reported that currently there is little consistency or standardisation of protocols, policies or guidelines across NSW to guide the treatment or follow up care for women who had sustained SPT during birth.

There was a discussion at a high risk meeting with the medical officers about where severe perineal trauma should be repaired and who should do what. There was no agreement whatsoever with them, nothing. Everybody does different things. (CMC)

CMCs believed that care for women who have experienced SPT varies and this variation ranges from local district policies and protocols to individual practitioner preferences on how immediate repair and long term care should occur. This was reported by participants from both metropolitan and rural LHD’s: “There’s not consistency. It’s more practitioner driven…”. The preferences of the individual medical practitioners were described as impacting upon the implementation of guidelines and policies. For example, it was reported that for one LHD a district wide policy existed in draft form, however this policy was unable to be progressed due to an inability to gain consensus from the area’s representative medical staff as to appropriate care guidelines.

Similarly, women described the challenges they faced in attempting to navigate treatment pathways, often reporting that the lack of identifiable and available services were inadequate in meeting their needs.

I felt like I didn’t have enough support, but I didn’t really know where to go to get it. I didn’t really know whether it actually existed. I just wished somebody would have just said, go to the GP. If there’s a problem, go and do something about it. (Indie)

Some CMC’s however described how a coherent policy guided how health professional provided care for women who had sustained SPT within their health service.

We have a policy. There is a policy in place and there is a pro forma that the midwives and doctors have to complete. (CMC)

#### ***Qualifications, skills, beliefs, and attitudes***

##### 

**Qualifications and skills** The CMCs described the level of skill and credentialing process required for health professionals to undertake repair of SPT to be inconsistent and unclear. In response to the survey question “Who is permitted to repair severe perineal trauma?”, participants indicated that the necessary qualifications to undertake repair ranged from resident medical officers RMO to specialist colorectal surgeons (Table 2). In individual health facilities where a credentialing process was reported to exist, the CMC’s were mostly unclear on what this process involved. It was reported that one tertiary level maternity unit had a clear credentialing process in place, whereby repairs were able to be conducted only by an obstetric and gynaecology (O&G) registrar in their third year of practice, a staff specialist or above. Some services had commenced a mentorship program for RMOs, however it was unclear what qualifications the mentor was required to have. Some facilities had no credentialing process at all:

There’s no level of credentialing for the registrars. I guess it’s left up to their individual level, just like the midwives are – “I don’t want to do this repair because it looks too big”. I’m hoping that’s what’s happening there. (CMC)

The CMC participants also expressed concern over inconsistencies around training staff to undertake a comprehensive perineal assessment following a vaginal birth. It was reported that junior staff or health professionals without current perineal assessment skills, may incorrectly diagnose the grade of perineal trauma, this may result in an inadequate repair being performed:

If the midwife is in there and she doesn’t recognise that it’s a third degree tear, that’s not going to get picked up…she’s not going to get anybody in there to do it. (CMC)

The participants discussed the roles that registrars, obstetricians and colorectal surgeons played in SPT repair. Some participants described a conflict that existed between the role and responsibilities of the registrar and colorectal surgeon: “…because there are obstetricians or VMO’s [Visiting Medical Officers] that get upset when the registrar calls in the colorectal surgeon”.

In contrast, other services described a collaborative process that had been established between the obstetricians, registrars and colorectal surgeons in identifying and repairing SPT but they were in the minority:

If they’re anything more than a 3A tear, then they [registrar] notify the colorectal surgeon. The colorectal surgeon is actually in theatre when they initially assess the tear, prior to deciding whether it’s worse than a 3B or whether it’s a four[th]. They actually work together. It’s a really, really good collaborative agreement. (CMC)

Within some LHDs the CMC representatives reported a concern at the lack of diagnostic facilities and therefore the accuracy of perineal assessment:

I’ve heard a colorectal surgeon talk about his “magic finger”. They don’t have manometry in hospital. They don’t have pudendal nerve testing and they don’t have endoanal scans specifically for women. So they’re not really assessing it properly at all. (CMC)

Some of the CMC participants believed that the inconsistencies in assessment, evaluation and repair were a result of perineal trauma not being seen as an obstetric priority and therefore standardisation of care and training was not seen as important: “There’s very little clinical review of particular cases. It’s not seen as something that they need to review. I think that needs a discussion as well”. (CMC)

In response to this, one service that conducted an audit and identified incorrect diagnosis contributing to increasing SPT statistics, introduced a policy stating that two health professionals were now required to review each women with perineal trauma (second degree tear or above), to increase the likelihood of correct identification and repair.

##### 

**Beliefs about the cause of SPT** In both the survey responses and the discussion, the majority of CMC participants indicated that they believed the incidence of third and fourth degree perineal trauma was increasing, and in response to this participants indicated that at a number of health facilities internal audits had been undertaken to identify the reasons for increased reporting (Table 2). On the survey, CMCs were asked to rank in order from 1 to 10 what they believed was the most significant contributor to the incidence of SPT, with position for second stage being highlighted as the most significant cause, followed by instrumental birth and episiotomy (Table 3).

Many of the women who participated in the interviews spoke of strategies they used, or had heard of, during the antenatal period, to minimise the risks of sustaining perineal trauma. These strategies included the use of oils and massaging their perineum to make the area more flexible for birthing, while one participant described using a device to stretch the perineum. Women reflected on these strategies, often describing that they had let themselves tear by forgetting or neglecting to perform perineal massage consistently during their pregnancy which resulted in them sustaining SPT. Other women stated the perineal trauma was due to a defect in their anatomy: “I just wasn’t stretchy enough and that’s why I tore.” (Asha). Further, women described that they felt incorrect pushing techniques, the use of instruments during the birth, and a rapid birth contributed to perineal trauma.

##### 

**The attitudes of health professionals** For women who have sustained SPT, they were less concerned about the method of perineal repair that was performed, and more concerned with how they were cared for during this time. During the suturing process, the women recall clearly the facial expressions, words and phrases that the midwives and obstetricians used during the suturing process. The women interviewed described how staff spoke “around them” and “about them”, often not speaking directly to the women, and this resulted in women feeling vulnerable and exposed during the procedure.

I got embarrassed at one point, I’m just thinking I can’t believe I’m just lying here like this and they’re having a little discussion about what’s going on. They didn’t discuss it with me at all. Maybe they just thought I wasn’t worth it. But maybe they think well they don’t understand it anyway so what’s the point? (Scarlett)

#### ***Falling through the gaps***

The participants described that the care that women received in both the immediate and long term postnatal period varied amongst LHD’s and individual practitioners. The majority of services provided information leaflets outlining perineal care to women prior to discharge. In response to the survey question: “Is there a follow up clinic available that women are referred to?”, ten participants responded with ‘yes’, three with ‘no’, and one participant was ‘unsure’. Some of the services offered a range of counselling services for women who had sustained SPT, other services reported that there were no routine postnatal support pathways in place.

The referral process for women to access follow up anal sphincter assessment was also seen to be practitioner dependent: “So someone like a specialist obstetrician may keep the women to review himself and, maybe, other areas where there’s GP obstetricians, they might refer through… ” (CMC)

In preparation for discharge from hospital, the level of support and information provided to each woman appeared to vary based upon the individual care provider, model of care, and health service. While the focus of the practitioners was on the care that was provided to women within the health service, for the women who were interviewed, being discharged from care presented new and unfamiliar challenges.

They just checked the stitches, made sure they were clean, made sure they were dry, but they didn’t explain any ongoing process…they just said if you have any problems go to your doctor....Nobody explained anything. (Matilda)

At discharge, while some participants described receiving referrals for an endoanal ultrasound and receiving community midwifery support, other women reported that they received no support.

The endoanal ultrasound is meant to take place 5-6 months after birth. I just received a letter today stating that the appointment has been cancelled and rescheduled for 20 months after the birth. I haven’t experienced any problems (that I’m aware of, anyway)….however it’s very worrying that there is a 20 month wait for this follow up scan. (Indie)

For many women the level of support following SPT was inadequate in addressing their needs. Women described feeling confused and unsure as to when and where to seek support, particularly for women experiencing symptoms such as urinary incontinence, perineal pain and urinary retention. As they struggled to understand what degree of perineal pain and symptoms were normal and not normal following birth, they spoke of the importance of the role of health professionals in providing appropriate care, information, and reassurance to provide support in the postnatal period.

I didn’t know that it wasn’t right. I guess, you rely on the health care professionals to tell you. This is actually a pretty big thing that you’ve had, and this is the follow up you need, and this is what we need to do, and if you’re in pain let me know and I’ll get you something. (Chloe)

Support for women experiencing a subsequent pregnancy and birth appeared to vary across the NSW. CMCs reported that while some facilities offered no clear follow up services and subsequent birth planning and support for women who had sustained SPT during a previous birth, other facilities offered comprehensive support services. This appeared to be driven by motivated midwives:

It’s recognised at booking in that these women have got issues [as a result of SPT] - they’re just [scared of] the next birth and they’ve taken, maybe, five, six years even to get pregnant. So we meet and go over the last birth, it’s usually a debrief about the birth. Then there’s no set guidelines to what the birth will be. It depends on what experience was the last birth. That’s the way I make the plan, together in consultation with our clinical director. (CMC)

When asked to describe the general recommendations given to women in planning subsequent births, responses varied depending upon the opinion and preference of the individual medical officer, and the degree of ongoing morbidities experienced by the woman. For the women in this study, the decision making process for planning subsequent modes of birth was complex, with the participants describing how their decision was influenced by the advice they received and how much trust they placed in the health care provider:

The doctor that I saw when I was pregnant said you don’t need a caesarean. I said it’s really quite a sensitive subject and I really don’t want to have to explain but I think you should understand by me saying leaking and fistula. He said just don’t have a big baby this time. He said don’t eat – I swear to God – he said don’t eat so much. You won’t put on so much weight, you’ll have a smaller baby, you won’t tear. I just thought, that is the stupidest thing I have ever heard. (Lola)

#### ***Caring for women who have sustained SPT***

How women were cared during the perineal repair process and in the postnatal period was a topic of concern for both the CMCs and the women. There were three identified areas of concern: the models of care within which women were cared for, whether the provision of postnatal care was focussed on the wellbeing of the newborn to the detriment of the new mother, and the provision of services for women who reside in rural and remote locations.

##### 

**Continuity of carer** During the discussion, continuity of carer was considered important for women who have sustained SPT during birth. It was believed that this would provide a level of consistency and be of value to women throughout any follow up consultations or diagnostic process:

The woman possibly has symptoms – she should have an open door if she’s got problems, to come back, so that she can be assessed by a midwife. Then, if there are significant issues, then it can be – some consistent care. (CMC)

Similarly, women described the value of receiving personalised, compassionate care from a known care provider, and the importance of receiving honest and accurate information communicated in a consistent and sensitive manner.

I really like that idea with the one midwife/one person – you know, you get a chance to iron out all the little crinkly bits before… it’s this whole womanhood, sisterhood, thing that I wanted to be a part of. (Ava)

The level of trust that existed between a woman and her health care provider appeared to impact upon whether or not the women felt in control, and therefore able to advocate for what they felt was important for themselves and their newborn. For women, the location that perineal repair was performed was an important consideration, and if given the choice of location the majority of women declined going to theatre due to their concern about leaving their newborn.

…they did say, oh we’re going to have to take you away for stitching. I’m like what are you talking about taking me away? I was like, If I’m going there, where’s my baby going to be? Oh well we’ll take her to the nursery and I’m like, hang on, how long will I be gone for? I was like, whoa just back up a minute and I said Well why would you think that I would want to do that? I’ve just had my baby. (Grace)

The decisions that women made when considering location of repair were further influenced by the level of trust they had in their care providers and in the health care system. For women who were given a choice on where the repair would occur, those who trusted their care providers and trusted that their newborn would be cared for appropriately followed the advice they were given. This was particularly noted for women who had continuity of care throughout their pregnancy, labour and birth.

So they asked whether I wanted to do it just under an epidural or whether I wanted to go under a general anaesthetic. I said I didn’t really care either way. But then a doctor came in and said “No, you need it under general anaesthetic, it wouldn’t be pleasant or easy to do it under an epidural, it would be a bit too traumatic”. I was fine with that. So [the new baby] went off with Daddy, and I got wheeled off into surgery. (Sophie)

In contrast, the location where the perineal repair took place appeared to be influenced by both LHD policy and practitioner preference. It was reported in both the discussion and survey that the majority of fourth degree tears were repaired in theatre while the repair of third degree tears occurred either in theatres or in the room that the woman had birthed in. This decision was reported to be based upon both availability of theatres, staff, and individual preference of the health professional performing the suturing:

Fourth degree tears always go to the operating theatres. As far as third degree tears, for a number of years they need to go to operating theatres. The medical officers will not agree, they think they’ve done it for years and they can go ahead and keep on doing it, exactly where it is, because they think it’s perfectly alright. (CMC)

##### 

**Woman centred or baby centred care?** Women felt that following the birth, the focus of the health professional moved from the woman to the wellbeing of the newborn baby, and as a result the information women received focussed upon the care of the newborn with little concern as to the wellbeing of the new mother. Receiving honest and accurate information was seen as being of particular importance during the suturing process and immediate postpartum period. Women also described the need for health professionals to be mindful of the language that they used, and their facial expressions, when caring for women undergoing assessment and suturing of SPT.

They don’t have to say anything, but if you look at someone you can tell what they’re feeling. I mean you don’t work in a factory, you’re not sitting there sewing a little jacket, you’re helping somebody birth a child. (Matilda)

It was seen as important to the women that more of a focus was given to the health of the mother, including providing information on how to care for the perineum following discharge, any potential symptoms and morbidities that can occur as a result of SPT, and what women should do if any of these were to occur. Women reported that receiving this information as a leaflet, booklet or online resource would be beneficial as it could be reviewed when they were ready, and would also be easily accessible by their partner.

It would be good to have something if you did have questions to look through. You know they teach you how to wash the baby, and there’s all sorts of diagrams –but there’s nothing about aftercare for the mother except keep it clean, and make sure you can go to the toilet. (Ava)

Following discharge from the hospital, women described a need for a postnatal appointment conducted earlier then 6 weeks for a review of the perineal trauma, at this time women felt they would also have an opportunity to debrief, ask any questions and be provided with clear treatment pathway options if ongoing support was required. The women felt that ongoing support through a support group facilitated by a health professional would be beneficial as women worked to understand the physiological and psychological impact of SPT.

##### 

**Adding complexity to the picture: services in rural NSW** Support for rural women was described as being underfunded and poorly structured which resulted in inconsistent follow up with little consideration of the distances women need to travel to access care. For families living in rural and remote locations, it was acknowledged that women who required follow up care were faced with additional expenses associated with accessing follow up care, such as travel and accommodation costs, and this was reported as another reason why women were less likely to seek follow up care.

Anecdotally I would say that a lot of those women don’t take that up (referral to a continence clinic) because they don’t live locally. When you’re looking within our area the women are travelling up to 400 and 500 kilometres to get that follow up. It’s only if they develop ongoing problems that they develop a need, eventually, to some follow up. (CMC)

For immediate repairs of SPT, a lack of rural based services and minimal staffing means that for some women they require transfer to the closest tertiary facility which can occur by ambulance or using their family car. For repairs on site, repairs occur as a result of collaborative efforts by GP specialists (obstetricians, anaesthetists, surgeons) who combine specialities and conduct the perineal repair. Follow up care was reported as “variable” depending upon the location and availability of skilled professionals.

Over a very large geographic area it’s very hard to standardise practice or to track what kind of counselling or debriefing the women might get. It will entirely depend on which care provider they see. The advice they get in terms of the next pregnancy is going to be dependent on those individual care givers. (CMC)

During the interviews with women, there were no participants who resided in a rural location, therefore this perspective is limited to that of the CMCs.

#### ***Gold standard care: how would it look?***

The CMC’s who participated in the discussion group clearly identified the structure and delivery of a service that would provide a comprehensive model of care to women who sustained SPT during childbirth. This was determined as important, as when asked during the discussion group if the CMCs felt that the incidence of SPT was increasing, the majority response indicated that they felt the incidence was increasing across NSW. In response to the survey question: “Rank in order from 1 to 10 (1 being most significant, 10 being least significant) what you feel contributes to the incidence of severe perineal trauma”, the most significant cause was identified as the position for second stage. The second most significant cause identified was instrumental birth (Table 3). The majority of the group identified that the key element in achieving a comprehensive level of state-wide care is consistency and standardisation of assessment, repair, treatment and ongoing care for women who sustained SPT. The implementation of mandatory reporting of cases of SPT including documentation of the degree of trauma, the method of suturing and the location at which the repair takes place, would facilitate clinical review, staff development, and the ability for audits to be conducted within the health services. It was suggested that this could be achieved through perineal repair training programs and an associated credentialing system for all health professionals who care for women who have sustained SPT, and the establishment of state-wide and national policies to guide standardised management across all health services.

I think consistency and standardisation of everything. That starts from all the psychosocial stuff, so that needs to be considered. Then there needs to be consistency with everybody; so the skills of identification, the credentialing of the people most appropriate to repair, even the materials. I mean the evidence is out there that says all that. (CMC)

In response to the survey question “If there was anything that you could incorporate into your service for women who experience SPT what would it be?” the CMCs prioritised the importance for health professionals to be competent in accurately assessing and repairing perineal trauma. Further, in the survey responses the CMC participants identified the importance of providing women who sustain SPT with a comprehensive physiological follow up including a consultation with allied health specialists, including a physiotherapist and colorectal surgeon, and associated testing to determine the existence of any anal sphincter defects. Ideally these services would be offered within a specialist pelvic floor clinic with a known care provider where women would be provided with consistent evidence-based information. It was determined that this service would provide ongoing support for women through clearly structured treatment pathways. The group also identified the benefits of establishing such a service for women contemplating, or experiencing, a subsequent pregnancy and birth following SPT:

I think for the next birth it should be that they come to a specific clinic and see a consistent practitioner in that clinic – either a midwife or an obstetrician – so that the birth plan can be formulated for them. (CMC)

It was identified that this support was also required for women who resided in rural and remote locations, ideally with the establishment of a multi-disciplinary team that would be made available for women who required assessment, support and ongoing care.

## Discussion

This study reports on CMCs and women’s description of health service provision for women who have sustained SPT following vaginal birth in NSW. It was apparent that there was a patchwork of policy and processes that were identified by both CMCs and the women as problematic. While the CMC’s were focussed on the technicalities of perineal repair and the processes of service provision, and women who have sustained SPT were more concerned with how they are cared for and treated by health care professionals, the CMCs clearly identified and were concerned about the impact of the “patchwork” system on the care provided to women.

### Risk factors for severe perineal trauma

Risk factors during the antenatal period associated with an increased incidence of SPT include parity, maternal age, ethnicity, nutritional status, fetal weight and abnormal collagen synthesis [5,28]. Intrapartum risk factors include medio-lateral and midline episiotomy, instrumental birth using ventouse and/or a forceps delivery, a prolonged second stage of labour, and the birth position adopted by the woman during second stage [29-33]. The CMCs who participated in this study identified antenatal and intrapartum risk factors for SPT, identifying the position for second stage as the factor most likely to contribute towards a woman sustaining SPT. In addition, instrumental birth and episiotomy were identified as intrapartum risk factors, which correspond with what is currently known about risk factors for SPT [4,5].

### Suturing the perineum – the importance of compassionate care

Research reporting upon the repair of SPT focusses on the outcomes of immediate and long term morbidities, however little research has examined women’s experience of perineal suturing [3,34,35]. While the CMC’s were focussed upon who was the most appropriate health professional to undertake the repair of SPT, the women who were interviewed focussed upon how much information they received regarding the repair, whether the actions of the health care professional undertaking the repair were appropriate, and how the process of suturing interfered with their ability to spend time and bond with their newborn.

Similar findings were reported in a prospective study conducted by Green et al. (1998) [36] who explored women’s experiences and expectations of childbirth, and their subsequent levels of satisfaction or dissatisfaction with the outcome [36]. Women who sustained perineal trauma describe perineal suturing as the “worst thing about birth” [36], p. 348, when reflecting upon the level of pain they had felt during the procedure, the lack of information they received about the repair, and being separated from their newborn baby during the procedure.

Some argue that health professional training is currently focused on the development of technical skill and less on the importance of providing woman-centred compassionate care [37]. The importance of compassionate care has been identified by the NHS Commissioning Board (2012) in the policy document “Compassion in Practice” (NHS Commissioning Board, 2012). This document identifies ‘compassion’ as one of the six fundamental ‘Value and Behaviour’ practices which sits alongside care, competence, communication, courage and commitment (referred to as the “6Cs”). Compassionate care is described as: “… how care is given through relationships based on empathy, respect and dignity - it can also be described as intelligent kindness, and is central to how people perceive their care. (NHS Commissioning Board, 2012, p. 13). The women in this study were able to clearly recall the way they were treated by health professionals throughout their labour, birth and postpartum period, and this treatment impacted upon the way women recovered from and processed their experience both negatively and positively.

### Clinician training

Little research has evaluated the training process for health professionals in accurately diagnosing and performing repair of SPT. It is recommended that a thorough assessment of trauma to the perineal, vaginal and anal sphincter regions immediately following birth is performed by an appropriately qualified health professional who is trained in pelvic anatomy and correct suturing techniques of the perineum [2,38-40]. Comprehensive examination is recommended both prior to, and following, suturing to ensure correct repair has been performed [3,38]. The CMCs who participated in the discussion group described the variation and inconsistencies across current NSW health services as to the minimum level of training required for health professionals to appropriately repair SPT. Perineal assessment performed by inadequately trained staff can lead to misdiagnosis of the degree of perineal trauma and consequently result in inadequate and inappropriate treatment, morbidities such as wound breakdown and abscess formation, and the development of co-morbidities such as recto-vaginal fistula [3,41-43].

Previous studies reporting upon the ability of health professionals to accurately identify and repair second degree perineal trauma have identified that the majority of midwives, trainee doctors and obstetricians describe their training and professional support as inadequate [37,44,45]. In a cross-sectional survey examining current practice, experience and confidence of obstetricians in the management and repair of SPT [46], the authors reported that the majority of obstetricians (95%) described themselves as confident in repairing SPT. Despite the level of confidence reported by the participants, the findings are similar to those of this study in demonstrating that there is a lack of consistency in the practice of SPT repair, with the participants identifying that a local guideline or protocol would be useful in guiding their practice [46].

### Postnatal referrals and pathways of care

The World Health Organisation state that despite the postnatal period being one of the most critical in contributing to optimal maternal and neonatal health, they describe the delivery of postnatal services as “… the most neglected period for the provision of quality care.” [47], p, 3. Bick (2005) states: “Despite pain being experienced by hundreds of thousands of women who give birth each year in the UK, and many more worldwide, identification and management of perineal morbidity…has not been a high priority. The postnatal management of more severe perineal trauma…has also been relatively neglected” [48], p. 113. This neglect is reflected in through the lack of current research that has been conducted into postnatal services.

The CMCs who participated in this study identified that postnatal services varied depending on the LHD and the practice of the individual health practitioners. This inconsistency in service provision was also reported by the women; while some women received postnatal support and referral to service and diagnostic examinations, other women received no follow-on care or support postnatally. Similar findings have been reported elsewhere, with women describing feeling let down by the health system as they try to manage ongoing morbidities with little to no support [9,10].

In this study women identified that the level of information and support they received following birth impacted upon their ability to identify what is normal and what is not normal during the postnatal period, and seek help accordingly. Consent advice guidelines distributed by the Royal College of Obstetricians and Gynaecologists (RCOG) highlight the importance of providing the woman with detailed information irrespective of the extent of the perineal trauma that has been sustained [49]. However, despite the availability of these guidelines, the women in this study reported that they were often provided with little or no information in relation to the degree of perineal trauma they had experienced or the process of the perineal repair therefore making it difficult for women to know what is normal and when to seek help.

### Gold standard care

It has been suggested that multi-disciplinary collaborative specialist clinics are required for women who sustain SPT, with the aim of providing comprehensive physiological and psychological support within the one facility [50-53]. Both the CMCs and the women who participated in this study identified the importance of support for women who sustained SPT, ideally provided by a known care provider. Additional benefits of establishing specialist perineal clinics are reported to include an efficient model of specialised health care, facilitating ease of access for women requiring services. This approach is supported by Chatoor et. al (2009) [51] who states: “The multidisciplinary team approach is a means of synchronising treatment between various specialities and streamlining the patient pathway [51], p. 614.

#### ***Limitations***

This study focusses on midwives experiences of caring for women who sustain SPT, however the CMC participants of this study come from metropolitan and rural NSW, Australia, therefore the context of these findings were specific to NSW and these findings may not be transferable to services in other States and Territories in Australia, or internationally [54]. While the context of these findings may be transferable, it would be important that the findings were related to the new context. For this study, a descriptive qualitative research design was chosen to facilitate a greater understanding of the topic under investigation, however due to the time limitation of the discussion group (one hour) and large number of CMC participants, this presented challenges in exploring in detail the CMC perspective of service provision [17,54]. Limitations that arose during the recruitment and interviews with women have been described in a previous publication [12]. No women who resided in a rural location were included. Strengths of this study include the use of an integrative approach which provided a comparative analysis of similarities and differences in the way women and CMCs view service provision for women who sustain SPT, this approach provides a deep understanding of complex phenomena as for this study [24,25].

## Conclusion

The findings from this study suggest that current health services in NSW represent a patchwork of service provision for women who have sustained SPT. This is characterised by a discrepancy between the services that are provided, and the needs of women. It appeared that women who experience SPT and associated postnatal morbidities seek compassionate and supportive care based upon a clear exchange of information, and this should be considered when reflecting upon the current health service design. This study highlights the benefits of establishing a comprehensive, multi-disciplinary collaborative specialist clinic to support women who experience SPT and associated morbidities, with the aim of providing comprehensive physiological and psychological support.

## Abbreviations

LHD: Local Health Districts, encompass all health facilities which operate within a geographical area determined by NSW Health; O&G: Obstetrics and Gynaecologic Registrar, A doctor who is undertaking specialist training in obstetrics and gynaecology; RMO: Resident Medical Officer, A doctor who has completed a medical degree and a 12 month internship, and are eligible for full registration with the medical board; VMO: Visiting Medical Officer, A doctor who provides medical services for hospital patients on sessional or fee for service basis.

## Competing interests

The authors declare that they have no competing interests.

## Authors’ contributions

HP participated in research design, data collection and analysis, design and drafting of manuscript as a component of a doctoral study. HD assisted with research design, data collection, review of data and manuscript drafts. VS assisted with research design, review of data and manuscript drafts. CK and AS assisted with identifying literature for the discussion, review of data and manuscript drafts. All authors read and approved the final manuscript.

## Pre-publication history

The pre-publication history for this paper can be accessed here:

http://www.biomedcentral.com/1471-2393/14/236/prepub
